# Sex-dependent vulnerability to early-life phthalates exposure and autism spectrum disorders: a systematic review

**DOI:** 10.3389/frcha.2026.1865057

**Published:** 2026-08-11

**Authors:** Ivana Bucaro, Paola Palanza, Davide Ponzi

**Affiliations:** Department of Medicine and Surgery, University of Parma, Parma, Italy

**Keywords:** autism spectrum disorder (ASD), early-life adversities, endocrine-disrupting chemicals (EDCs), phthalates, sex-dependent vulnerability

## Abstract

**Background:**

Autism spectrum disorder (ASD) is a neurodevelopmental condition characterised by restricted and repetitive behaviours (ICD-11) and severe and persistent difficulties in social interaction and communication. A marked male predominance has consistently been reported, with ASD being three times more common among boys than girls, although the biological mechanisms underlying this sex difference remain unknown. Physical and chemical stressors occurring during critical windows of development may alter foetal and infant physiology and interfere with neurodevelopmental trajectories. Among these stressors, endocrine-disrupting chemicals (EDCs), including phthalates, have been proposed as potential environmental contributors to neurodevelopmental vulnerability. In this systematic review, we examined whether early-life exposure to phthalates may be associated with sex-specific vulnerability to ASD and autistic traits.

**Methods:**

The systematic review was conducted in accordance with the PRISMA guidelines and followed a PECO framework. The target population included human children of both sexes, ranging from early childhood to adolescence. Exposures of interest included prenatal and/or early-life exposure to phthalates, assessed through biological matrices during prenatal and/or postnatal periods. Eligible outcomes included ASD and autistic-like traits assessed using validated diagnostic or screening tools. The review was registered in PROSPERO (CRD420261438630).

**Results:**

A total of 18 studies were included. Phthalates exposure was assessed across a broad developmental window, ranging from the preconception period to 8 years of age. Urinary biomarkers represented the primary method for assessing chemical exposure, while a smaller proportion of studies also relied on blood samples. Sample size ranged from 77 to 3,220, and the age at assessment of autistic traits or ASD-related outcomes ranged from 18 months to 15 years. The Social Responsiveness Scale (SRS) was the most frequently used instrument for assessing autistic traits, followed by the Autism Diagnostic Observation Schedule (ADOS).

**Conclusion:**

Prenatal exposure to phthalates, especially during early and mid-gestation, appears to be associated with subtle increases in autistic traits, with some evidence suggesting greater susceptibility among boys, particularly in relation to MBP, MEP, and DEHP-related metabolites. However, evidence remains limited and heterogeneous, and current findings require further confirmation.

**Systematic review registration:**

https://www.crd.york.ac.uk/PROSPERO/home, PROSPERO CRD420261438630.

## Introduction

Autism spectrum disorder (ASD) is a heterogeneous neurodevelopmental condition characterised by social communication difficulties and restricted, repetitive behaviours (1, 2). ASD is a common childhood disorder, that arise early in development, persists across the lifespan and affects approximately 1% of the population (3). Reported ASD prevalence has increased over time. Data from the Autism and Developmental Disabilities Monitoring Network report showed an increase from one in 150 in 2,000 to one in 44 in 2018 among 8-year-old children, and prevalence among boys remained substantially higher than that among girls (4–6).

Core symptoms in the social communication domain may include persistent difficulties in social interaction and reciprocal communication. These may involve reduced or atypical eye contact, a lack of response to one's name, limited use or absence of facial expressions, and delays or differences in early social engagement behaviours (e.g., interactive games) (2). Restricted and repetitive behaviours and interests associated with ASD can include repetitive or stereotyped use of objects or speech (including echolalia), insistence on sameness and adherence to routines, distress in response to minor changes, highly restricted or fixated interests, repetitive motor movements and atypical responses to sensory stimuli. Other commonly associated characteristics include delay in language, motor or cognitive development, as well as, attentional difficulties, epilepsy and other seizure disorders, atypical eating and sleep patterns, gastrointestinal symptoms, emotional dysregulation, anxiety or excessive worry, and altered fear responses (2).

The causes of ASD are multifactorial. Twin and family studies confirm that ASD is highly heritable, but they also indicate that non-genetic influences contribute significantly to the risk of ASD, particularly when they operate during sensitive windows of brain development (7–9). During gestation, the developing brain undergoes through a rapid, highly coordinated and overlapping sequence of developmental events. Early gestation is characterized predominantly by neuronal proliferation and migration, the second and third trimesters of pregnancy by neuronal differentiation, synaptogenesis, gliogenesis, and myelination, whereas the third and postnatal period are characterised by the refinement of neural circuits (10, 11). Many of the genes associated with ASD are expressed in the neocortex during prenatal development (12) and converge within broader foetal brain biological networks linked to these neurodevelopmental processes (13, 14). Although much of this genetic susceptibility is inherited, gene expression depends on endocrine signals that in turn depend on internal and external environmental factors. Perturbations of these developmental processes may alter the neural architecture and functional connectivity of the brain, thereby supporting a link between ASD susceptibility and early developmental stages.

### Sex as a biological vulnerability for ASD

ASD exhibits one of the most pronounced sex differences in prevalence among neurodevelopmental disorders. Although recent data show a reduction of the male-to-female ratio (15), the predominance of ASD diagnoses in males is well established (11, 16). With regard to inheritance of common genetic variants linked to ASD, males and females appear to have a similar level of risk (17, 18), suggesting that other factors must be involved in this sex-dependent vulnerability. On the one hand, the *female protective effect* (FPE) theory posits that girls require a greater mutational or environmental burden before meeting the diagnostic threshold for ASD. The FPE framework suggests that certain genetic or hormonal factors may protect neurodevelopment in girls from the effects of ASD-related risk factors (19, 20). On the other hand, the *prenatal sex steroid* theory of autism (21) suggests that elevated exposure to prenatal sex steroids, particularly foetal testosterone (fT), is a major risk factor for autism.

Support for the *prenatal sex steroid* theory is based on the observation that higher levels of sex steroids in the amniotic fluid are positively correlated with autistic traits and autism diagnosis (21–23) and on evidence of an increased likelihood of ASD diagnosis among women with polycystic ovary syndrome, a condition characterised by elevated testosterone levels, and among their offspring (24). As autistic traits are more frequently reported in males in the general population, the *extreme male brain theory* of ASD (25) proposes that autism represents an extreme manifestation of a male-typical cognitive profile influenced by the masculinizing effects of testosterone.

From early gestation onwards, sex steroids contribute to the organization of brain development (26, 27) regulate neuronal proliferation, and, androgens, specifically, influence the balance between excitatory and inhibitory neuron subtypes (28, 29). Individuals with ASD have been reported to exhibit early enlargement of the cerebral cortex (30), a higher number of cortical neurons, particularly in the frontal and prefrontal regions, reduced neuronal volume, and disorganised cortical lamination (31, 32). Although the exact mechanisms underlying the relationship between ASD-associated genetic networks and endocrine function remain unknown, sex steroids appear to influence genes and genetic pathways associated with ASD (33, 34).

### Biological sex, phthalates and ASD

The foetal brain is particularly vulnerable to environmental insults capable of disrupting hormonal homeostasis (11, 35, 36). Environmental exposures can influence foetal neurodevelopment partly by altering maternal immune activation, inflammatory cytokines signalling, placental function, glucocorticoid signalling, thyroid signalling, and sex-steroid signalling. These signalling systems are powerful regulators of foetal gene expression and may amplify, buffer or otherwise modify pre-existing biological vulnerability by modifying gene transcription and epigenetic regulation (11). Consistent with these observations, exposure to Endocrine-Disrupting Chemicals (EDCs) has been associated with ASD. EDCs can mimic or block hormones, or alter their synthesis, transport, and metabolism. They can cross the placenta and are detected in breast milk and infants' urine (37, 38). Animal models have shown that EDCs exposure during critical developmental periods can disrupt sex-steroid signalling and alter sex-specific patterns of emotional and cognitive behaviour (39).

With regard to the potential role played by environmental insults on ASD, particular attention has been directed toward phthalates. Phthalates are a group of non-persistent synthetic diesters of phthalic acid that are widely used as plasticizers in various products, ranging from industrial and housing products to cosmetics and food packaging materials (40). Because phthalates can leach, migrate or off-gas from finished products, human exposure is ubiquitous (40). Pregnant women, foetuses and young children are constantly exposed through food packaging, personal care products, household materials, medical devices, indoor dust and children's toys (41, 42). Phthalates can be categorized in two groups, according to their molecular weight. Common low molecular weight phthalates (LMW) include dimethyl phosphate (DMP), diethyl phosphate (DEP), di-n-butyl phthalate (DBP), and diisobutyl phthalate (DIBP), whereas high molecular weight (HMW) examples include di-2 ethylhexyl phthalate (DEHP) and di-isononyl phthalate (DINP). LMW phthalates are rapidly metabolized to form monoesters such as mono-ethyl phthalate (MEP), monobutyl phthalate MBP (or MnBP), whereas HMW phthalates are further transformed to oxidized metabolites such as mono-(2-ethylhexyl) phthalate (MEHP), mono-(2-ethyl-5-hydroxy-hexyl) phthalate (MEHHP), mono-(2-ethyl-5-oxo-hexyl) phthalate (MEOHP). Once in the body, phthalates can exert several biological effects by disrupting the hypothalamic-pituitary-gonadal, hypothalamic-pituitary-thyroid, and adrenal axes through their interaction with nuclear receptors, membrane receptors, intracellular signalling pathways, and gene expression (43–45).

Phthalate exposure has been associated with alterations in maternal steroid hormones concentration, including lower progesterone concentrations (46), higher oestrogens concentrations during early gestation, and lower testosterone concentrations during mid-to-late pregnancy following DEHP exposure (47, 48). Depending on the specific compound, phthalate and their metabolite, may exhibit weak estrogenic activity or antiandrogenic activity (43, 49) and may disrupt the normal process of brain masculinization (39, 50, 51). Prenatal phthalate exposure in rodents has been associated with neuronal degeneration; reduced neuronal and synaptic density in the prefrontal cortex; impaired cognitive flexibility; inhibition of cerebellar granule cell proliferation; disruption of foetal thyroid hormone signalling; altered aromatase activity; increased oxidative stress; and persistent emotional and cognitive impairments later in life (52–55). Importantly, many of the effects reported in animal studies are sex- and time- dependent (39). These findings suggest that early-life exposure to phthalates may differentially influence male and female neurodevelopment, highlighting the importance of considering sex as a key biological modifier of susceptibility to adverse neurodevelopmental outcomes.

### The present review

Prenatal exposure to phthalates has been associated with disruption of neurodevelopmental processes, difficulties in cognitive and psychomotor development and behavioural problems (43). However, the broader epidemiologic evidence linking phthalates to neurodevelopment remains generally slight or indeterminate (56). A previous systematic review found limited but concerning associations between prenatal exposure to phthalate and ASD (57), while more recent cohort-based reviews have reported positive associations between specific phthalates metabolites, including MBP and mono-3-carboxypropyl phthalate (MCPP) (58, 59).

The main contribution of the present review is its focus on sex-specific vulnerability in the association between phthalate exposure and ASD-related outcomes. To provide a clear and comprehensive overview of our findings, we first present the overall associations between prenatal and early-life phthalate exposure and ASD or autistic traits, organized according to the timing of exposure. Prenatal exposure is considered by trimester, followed by postnatal exposure. We then examine the same body of evidence from a sex-specific prospective, highlighting whether and how included studies have addressed potential differences in susceptibility between males and females. This sequential presentation was adopted to facilitate interpretation of the available literature.

## Materials and methods

This systematic review was conducted in accordance with the PRISMA (60) guidelines, followed a PECO framework and was registered in PROSPERO (CRD420261438630). The four PECO components were defined as follow: (1) *populations of interest*: children of both sexes, with an age range spanning from early childhood to adolescence; (2) *exposures:* prenatal and postnatal exposure to phthalates measured directly in biological samples, (3) *comparators*: participants with lower or different levels of exposure in cohort studies, and control group in case-control studies; and (4) *outcomes*: a clinical diagnosis of ASD or autistic traits assessed using validated diagnostic or screening instruments.

### Search strategy

We searched PubMed, Web of Science, Scopus, and Google Scholar using appropriate Boolean operators. The last literature search conducted on February 23, 2026. Studies were included if they: (1) used an designs, including cohort, case-control, or cross-sectional studies; (2) included children of both sexes;(3) examined prenatal and postnatal exposure to phthalates; (4) assessed exposure directly in biological sample (blood, urine, cord blood, breastmilk); (5) assessed ASD or autistic traits using validated diagnostic or screening tools; and (6) were published in English between 1995 and 2025. Animal studies were excluded.

**Figure 1:**
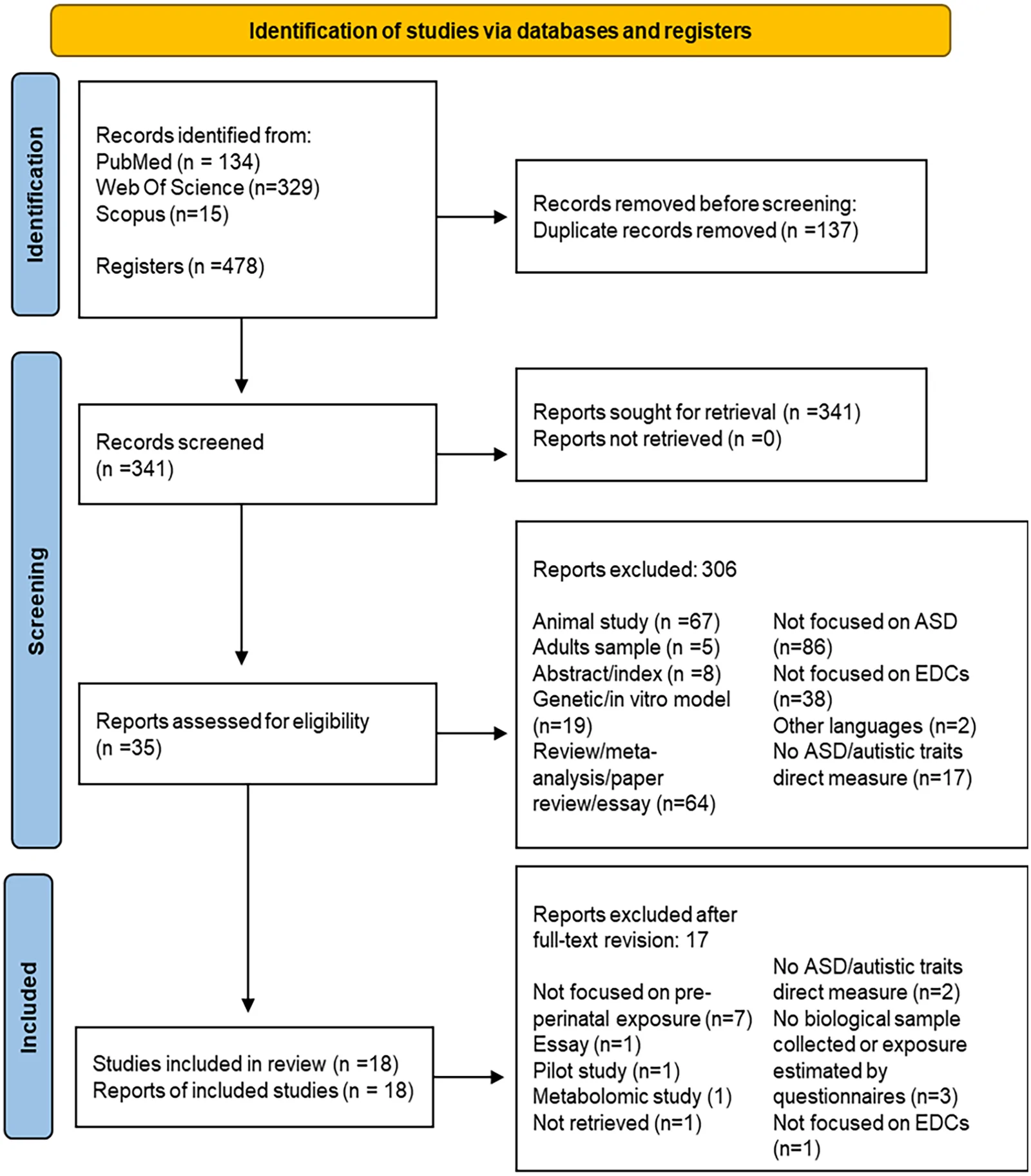
EDCs study selection process. PRISMA 2020 flow diagram for new systematic reviews which included searches of databases and registers only.

The search strategy included specific terms related to autism spectrum disorder and phthalates (“autism spectrum disorder” OR autism OR “autistic traits”) AND (phthalate* OR “phthalates” OR “DBP” OR “BBP” OR “DEHP” OR “MBzP” OR “MEHP”). Duplicate records were removed using Zotero. Following duplicate removal, a single reviewer conducted the first title and abstract screening according to predefined inclusion and exclusion criteria. Full-text articles were then assessed for eligibility by the same reviewer. To improve the reliability of the selection process, eligibility decisions for the final set of potentially eligible studies (*n* = 35), were discussed with a second reviewer. Consensus was reached on the final inclusion of 18 studies in the synthesis. The study selection process is illustrated in Figure 1.

From each study the following data were extracted: (1) study characteristics, including author, year, country, study design, period of the study; (2) participant characteristics, including sample size, age range/age at diagnosis, sex distribution, type of population; (3) exposure characteristics, including the chemicals or metabolites assessed, the exposure window and the biological matrix or matrices used; (4) outcome measures, including an ASD diagnosis or autistic traits; (5) effect estimates, including odds ratios (OR), risk ratios (RR), hazard ratios (HR) and regression coefficients; and (6) covariates and potential confounders considered in the analyses, including maternal age, maternal metabolic conditions during pregnancy, socioeconomic status, birth weight. Reported effect estimates included ORs, RRs, HRs and regression coefficients, with their corresponding 95% confidence intervals. Studies were grouped and synthesized according to exposure window (e.g., prenatal, gestational trimester, postnatal, childhood exposure).

### Risk of bias assessment

The methodological quality and the risk of bias were assessed using an adapted version of the Newcastle-Ottawa Scale (NOS) in Systematic Reviews (61). The NOS was modified to account for the specific characteristics of the included study designs and the exposure/outcome definitions relevant to the present review. The following domain were evaluated: (1) *representativeness of the sample*, defined as the inclusion of participants with a confirmed ASD diagnosis or the presence of autistic traits within the study population; (2) *clarity of ASD definition or autistic traits*, operationalised as the use of validated diagnostic instruments or psychometric tools; and (3) *appropriateness of the control/comparison group*, assessed in terms of baseline comparability between groups, including sex distribution and other relevant sociodemographic characteristics (e.g., child age, parental age, parental education, socioeconomic status, household income, ethnicity, geographical area, and recruitment setting); (4) *exposure adequacy*, evaluated based on whether prenatal exposure was comprehensively assessed across all trimesters. For cohort studies, the outcome domain additionally included the adequacy of outcome assessment, the length of follow-up, an acceptable loss to follow-up and the consistency of exposure assessment across groups. For case–control studies, the exposure domain further considered the appropriateness of the exposure assessment methods and comparability between cases and controls. Overall risk of bias was classified as low (7–9 stars), moderate (4–6 stars), or high (0–3 stars), based on the total NOS score. The risk-of- bias assessment was initially performed by one reviewer and subsequently discussed and refined with a second reviewer. Any discrepancies were resolved through consensus. The detailed risk-of-bias assessment is reported in Supplementary Table S1 and represent the final agreement between the two reviewers.

## Results

A total of 478 records were identified. After removing 137 duplicates using Zotero, 341 records remained. Following the first title and abstract screening, 306 records were excluded for the following reasons: animal studies (*n* = 67), studies involving adult samples (*n* = 5), abstract or index records without eligible full texts (*n* = 8), genetic or *in vitro* model studies (*n* = 19), review or meta-analysis (*n* = 64), studies not focused on ASD (*n* = 86), studies not focused on phthalates (*n* = 38); studies not published in English (*n* = 2); and studies that did not include a direct measure of ASD or autistic traits (*n* = 17).

A total of 35 records were subjected to full-text assessment, of which 18 were included and 17 were excluded. Reasons for exclusion were as follows: the study not assess phthalates exposure during an eligible developmental window (*n* = 7), the article was an essay (*n* = 1), a metabolomic study (*n* = 1) or a pilot study (*n* = 1); the full text could not be retrieved (*n* = 1); the study did not include a direct measure of ASD or autistic traits (*n* = 2), biological samples were not collected or exposure was assessed through questionnaires rather than biological measures (*n* = 3); and the study did not focus on phthalates (*n* = 1) (See Figure 1). A summary of the characteristics of included studies is presented in Table 1.

**Table 1:** Summary of the characteristics of included studies examining environmental chemicals exposure and ASD outcomes, including sex-specific effects.

Abbreviation	Full name	Method of administration	Description
ADOS	Autism diagnostic observation schedule	Interactive observation by trained clinician	A semi-structured assessment using various activities allowing observation of social and communication behaviors related to the diagnosis of pervasive developmental disorders.
BASC-2	Behavioral assessment system for children, 2nd edition	Parent/teacher questionnaire and direct observation	A comprehensive, multi-method and multi-informant assessment system designed to evaluate the behavioral and emotional functioning of children and adolescents.
CABS	Clancy autism behavior scale	Parent or teacher questionnaire	A 14-item screening tool identifying young children with autism by assessing behavioral manifestations, exploring difficulty in social interaction, lack of fear of danger, resistance to change, repetitive behavior, lack of eye contact, and unusual attachment to objects.
CHAT	Checklist for autism in toddlers	Parent questionnaires and direct observation	CHAT: Screening tool at 18 months combining 9 parent-report items and 5 direct observations (e.g., eye contact, joint attention, pretend play); high specificity (∼98%) but low sensitivity (∼38%), mainly identifying more severe early cases.
M-CHAT	Modified checklist for autism in toddlers	Parent questionnaire	M-CHAT: 23-item parent questionnaire for children 18–24 months; includes critical items indicating autism risk; developed to improve sensitivity compared to CHAT.
SCQ	Social communication questionnaire	Parent questionnaire	A total of 40 yes-no questions to evaluate communication skills and social functioning in children who may have autism spectrum disorders.
SRS	Social responsiveness scale	Parent questionnaire	A 65-item rating scale that measures the severity of autism spectrum symptoms by assessing social impairments, social awareness, social information processing, capacity for reciprocal social communication, social anxiety/avoidance, and autistic preoccupations and traits.

### Assessment tools for autism symptoms and diagnosis

Across the included studies, the *Social Responsiveness Scale* (SRS) (62) emerged as the most frequently used instrument for assessing autistic traits. The SRS is a standardised questionnaire completed by parents, teachers or caregivers and is designed to assess the severity of autism-related characteristics in children and adolescents aged 4–18 years. It consists of 65 items and evaluates behaviour in naturalistic settings, making it useful for screening and for supporting diagnostic assessment. The SRS provides a dimensional measure of autistic traits across five key domains: Social Awareness (referring to the ability to detect and perceive social cues), Social Cognition (referring to the capacity to interpret social information), Social Communication (encompassing reciprocal social communication skills), Social Motivation (referring to motivation to engage in social interactions, including social anxiety or avoidance), and Autistic Mannerisms (encompassing restricted interests and repetitive or stereotyped behaviours). Higher scores indicate greater levels of autism-related behaviours. Others assessment tools identified in the included studies were observational instruments, screening tool and behavioural questionnaires.

The *Autism Diagnostic Observation Schedule* (ADOS) (63) is a standardised, semi-structured interactive observation administered by trained clinicians. It involves interactive activities designed to elicit and directly observe social interaction, communication, play, and restricted or repetitive behaviors.

The *Behavioral Assessment System for Children*, 2nd edition (BASC-2) (64) is a comprehensive, multi-informant assessment system designed to assess behavioural and emotional functioning across home and school context. It incorporates reports from parent, teachers, as well as self-report measures where applicable.

The *Clancy Autism Behavior Scale* (CABS) (65) is a 14-item parent- or teacher- reported questionnaire designed to assess behavioural features associated with autism, including difficulties in social interaction, lack of fear of danger, resistance to change, repetitive behaviour, reduced eye contact, and unusual attachment to objects.

Screening instruments used for early detection included the *Checklist for Autism in Toddlers* (CHAT) (66) and the *Modified Checklist for Autism in Toddlers* (M-CHAT) (67). The CHAT combines parent-report items with direct clinician observation to assess early social-communication behaviours such as eye contact, joint attention, pretend play, pointing, and the ability to build a block tower. The M-CHAT is a 23-item parent-report questionnaire focusing on early autism-related behaviours. Like the CHAT, it includes critical items, the failure of which indicates increased likelihood for autism spectrum conditions.

The *Social Communication Questionnaire* (SCQ) (68) is a 40-item parent- or teacher- reported screening questionnaire assessing social interaction, communication, restricted and repetitive behaviours. Scores above the established threshold indicate an increased likelihood of an autism spectrum condition and suggest the need for further diagnostic evaluation. The validated psychometric tests used in the included studies to measure autism symptoms and for diagnosis are summarised in Table 2.

**Table 2:** Validated psychometric tests to measure autism symptoms and for diagnosis reported in studies included.

Author, year, country, study design	Sample size, population and period of study	Type of EDC	Biological matrix and timing of exposure	Outcomes, assessment tools and age at assessment	Main findings	Sex-specific effects
Alampi et al. (52) Canada Cohort	*N* = 478 mother–infant dyads (*n* boys = 235; *n* girls = 243) Maternal–Infant Research on Environmental Chemicals (MIREC) General population 2008–2011	Phenols (BPA) Phthalates (MBP, MBzP, MCPP, MEHHP, MEHP, MEOHP, MEP) Metals (arsenic, cadmium, lead, manganese, mercury)	Maternal blood/plasma/urine 1st trimester of pregnancy (6–13 weeks’ gestation)	*Autistic-like behaviors* SRS-2 (3–4 years)	Higher levels of lead, cadmium, bisphenol-A, mono-3-carboxypropylphthalate (MCPP), mono-butyl phthalate (MPB) associated with higher mean SRS scores. *Adjustment confounders* Child sex, folic acid supplementation, caregiver environment score, household income, relationship status, maternal ethnicity, age, education, parity, and city of residence.	Boys: stronger associations for MBP, MEP, and MBzP Girls: stronger associations for oxychlordane and trans-nonachlor. *Differences in associations between the sexes were pronounced at the 90th percentile of SRS scores.*
Al-Saleh et al. (58) Saudi Arabia Cohort	*N* = 456 children (*n* boys = 334; *n* girls = 121) *N* children (full assessment) = 125 *N* ASD = 51 (*n* ASD = 30; *n* girls ASD = 20 Saudi Early Autism and Environment (SEAES) General population 2019–2022	Phenols (BPA) Phthalates (MEP, MiBP, MBP, MBzP, MECPP, MEHHP, MEOHP)	Maternal urine 1st, 2nd, 3rd trimester of pregnancy	*Neurodevelopment* ASQ-3 (6, 12,18 months)—*Autistic-like behaviors* M-CHAT (18 months)	Associations between ASQ-3 scores and phthalate metabolites/BPA observed across the three trimesters, mainly in communication, gross motor, and problem-solving domains. Higher MBP, MiBP, and BPA 1st trimester exposure linked to decreased gross motor scores. MiBP 3rd trimester exposure and BPA 2nd trimester exposure associated with lower personal-social scores. MEP and low-molecular-weight phthalates linked to improved communication scores. Some 1st trimester exposures (MEP, MEOHP) associated with lower odds of a positive M-CHAT screen, while 1st trimester MBP exposure associated with potential increase in risk. *Adjustment confounders* Maternal weight gain, gravidity, education level, health conditions, medications, supplements, herbal remedies, pregnancies assisted by IVF or infertility drugs, exposure to environmental tobacco smoke, and the infant's age and gender.	Boys: stronger adverse associations in problem-solving and social domains. Girls: more affected in gross and fine motor scores. 2nd trimester BPA exposure associated with lower personal-social scores in both sexes 1st trimester MiBP exposure affected personal-social scores only in girls. *ASQ is not a direct measure of autistic like behaviors, but lower score in personal social domain may indicate potential autism-like behaviors.*
Barkoski et al. (68) US Cohort	*N* = 207 children (*n* boys = 122; *n* girls = 85) *N* ASD = 47 (*n* ASD = 31; *n* girls ASD = 16 Markers of Autism Risks in Babies-Learning Early Signs (MARBLES) High-risk ASD population 2007–2014	Phenols (BPA, BPS, BPF, TCC, TCS) Parabens (BuPB, ETPB, MEPB, PRPB)	Maternal urine 2nd, 3rd trimester of pregnancy	*Cognitive development* MSEL (3 years)—*Autistic-like behaviors* ADOS (3 years)	Prenatal exposure to BPA and parabens not associated with the risk of ASD at 3 years old. *Adjustment confounders* Pre-pregnancy body mass index (BMI), peri-conceptional prenatal vitamin use, homeowner status, birth year, and child's sex.	No sex-specific effects observed.
Bennett et al. (64) US Case control	*N* = 627 children (*n* boys = 484; *n* girls = 143) *N* ASD = 224 (*n* boys = 181; *n* girls = 43) Childhood Autism Risks from Genetics and Environment (CHARGE) General population and ASD 2006–2017	Phenols (BP1,2,3,8, BPA, BPF, BPAF, BPB, BPF, BPP, BPS, BPZ, BUPB, DCP24,25, DHB34, ETPB, HP4, HEPB, OH4BP, OHETP, OHMEP, PCP, TCC, TCP245,246, TCS) Phthalates (MiBP, MCHP, MCHPP, MCINP, MCIOP, MCHHP, MECPP, MEHHP, MEOHP, MEP, MHXP,, MINP, MIPP, MMP, MBP, MOP) Pesticides (DMP, DEP, DEMTP, DETP, DMDP, DEDP) Trace Elements (As, Be, Cd, Mo, Tl, U)	Children urine 2–5 years	*Cognitive development* MSEL (2–5 years)—*Autistic-like behaviors* ADI-R (2–5 years) ADOS (2–5 years)—*Adaptive behavior* (2–5 years)	Phenols/Parabens: higher children's urinary concentrations of MEPB, DHB34, and PRBP were associated with increased odds of ASD. Mixture analyses: DEP, MEPB, PRPB, ETPB, and MCINP contributed to ASD risk, while MEPB, TCP246, DHB34, MCPP, and MEHHP contributed to developmental delay risk. Phthalates: Only higher MCINP levels associated with increased odds of ASD. Mixture effect analyses: positive associations with both ASD and developmental delay. *Adjustment confounders* Child's sex, year of birth, age in months at time of diagnosis, and race, as well as parental homeowner status, and maternal metabolic conditions during pregnancy.	No sex-specific effects discussed.
Braun et al. (69) US Cohort	*N* = 175 mother-infant dyads (*n* boys = 80; *n* girls = 95) Health Outcomes and Measures of the Environment (HOME) 2003–2006	Phenols (BPA) Phthalates (MEP, MiBP, MBzP, MCPP, MEHP, MEHHP, MECPP) PFAS (PFOA, PFOS, PFNA, PFHxS) OC pesticides (trans-nonachlor, DDT, HCB)	Maternal urine/blood 1st, 2nd, 3rd trimester of pregnancy	*Autistic-like behaviors* SRS (4–5 years)	Phthalates, BPA: No association with SRS scores at 4–5 years. Trans-nonachlor and p’p’-DDT: Higher maternal exposure associated with higher SRS scores. *Adjustment confounders* Maternal age at delivery, race, marital status, education, parity, insurance status, employment, household income, prenatal vitamin use, depressive symptoms, maternal IQ, tobacco smoke exposure, caregiving environment.	No overall sex differences found for phthalates, BPA, and BFR exposures. Girls: maternal exposure to HCB and trans-nonachlor was associated with higher SRS score.
Choi et al. (66) US Cohort	*N* = 110 mother-infant dyads (*n* boys = 69; *n* girls 41) *N* ASD = 25 (*n* boys = 19; *n* girls = 6) Markers of Autism Risks in Babies-Learning Early Signs (MARBLES) High-risk ASD population 2006–2015	Phenols (BPA, BPS, BPF, TCC, TCS) Phthalates (MEP, MiBP, MBP, MHBP, MBzP, MEHP, MEHPP, MEOHP, MECPP, DEHP, MCPP, MNP, MCOP, MCNP) PFAS (PFHxS, PFOA, PFOS, PFNA, PFDA, PFUA, PFDoDA, Me-FOSAA, Et-FOSAA)	Maternal urine/blood 1st, 2nd, 3rd trimester of pregnancy	*Cognitive development* MSEL (3 years)—*Autistic-like behaviors* ADOS (3 years)	Prenatal exposure to PFAS, parabens/triclosan and phthalates/BPA associated with increased ASD risk. *Adjustment confounders* Child sex, year of birth, maternal age, maternal race/ethnicity, parity, maternal pre-pregnancy BMI, maternal education, and homeownership.	No sex-specific effects discussed.
Day et al. (54) US Cohort	*N* = 501 mother-infant dyads The Infant Development and Environment Study (TIDES) General population 2010–2012	Phthalates (MEP, MBP, MiBP, MBzP, MEHP, MEOHP, MEHHP, MECPP, MCPP)	Maternal urine 1st and 3rd trimester of pregnancy (6–21; 24–42 week's gestation)	*Behavioral problems* BACS-2 (4–6 years) *Autistic like behaviors* SRS-2 (4–6 years)	Early prenatal exposure to phthalates (MCPP, MBzP, MEP) associated with increased autistic traits in both sexes. Weaker associations with behavioral and adaptive outcomes. *Adjustment confounders* Pre-pregnancy BMI, study centre, race, income, maternal education, child sex, parity, time of the day and gestational age at urine collection, self-reported alcohol and cigarette consumption during pregnancy, and maternal depression and stress.	Boys: late prenatal phthalates exposure (3rd trimester) associated with higher externalizing behaviors scores (BACS-2) Girls: early prenatal phthalates exposure (1st trimester) associated with worse adaptive skill scores (BACS-2)
Emma et al. (61) US Cohort	*N* = 271 mother-infant dyads Health Outcomes and Measures of the Environment (HOME) General population 2003–2006	Phthalates (MBP, MBzP, MECPP, DEHP, MEP, MiBP)	Maternal urine 2nd, 3rd trimester of pregnancy	*Autistic-like behaviors* SRS (4–8 years)	Mixture analysis: prenatal phthalate exposure *positive but non-significant* associated with SRS scores. Higher prenatal exposure to MBP, MBzP, MiBP, MCPP, and DEHP associated with increasing SRS *T*-scores, particularly at higher percentiles. *Adjustment confounders* Maternal age at delivery, maternal race/ethnicity, maternal parity, pre-pregnancy BMI, household annual income child sex at birth child age at SRS administration, and study site.	Mixture analysis: positive but non-significant associations between prenatal phthalates exposure and SRS score both in males and females. Similar associations for MBP, MBzP, MEP, and DEHP between boys and girls at lower SRS percentiles; stronger associations in boys at the highest percentiles
Emma et al. (61) US Cohort	*N* = 166 mother-infant dyads Early Autism Risk Longitudinal Investigation (EARLI) High-risk ASD population 2009–2012	Phthalates (MBP, MBzP, MECPP, DEHP, MEP, MiBP)	Maternal urine 1st, 2nd, 3rd trimester of pregnancy	*Autistic-like behaviors* SRS (3 years)	Mixture analysis: prenatal phthalate exposure *negative but non-significant* associated with SRS scores. Weak or negative associations or negative for MBP, MEP, and DEHP, and null for MiBP and MCPP across SRS percentiles. *Adjustment confounders* Maternal age at delivery, maternal race/ethnicity, maternal parity, pre-pregnancy BMI, household annual income child sex at birth child age at SRS administration, and study site.	Mixture analysis: negative but non-significant associations between prenatal phthalates exposure and SRS score both in males and females. No effect modification by child sex observed for MBzP, MiBP, MCPP, or DEHP.
Gao et al. (53) China Cohort	*N* = 3,209 mother-infant dyads (*n* boys = 1,639; *n* girls = 1,570) Ma’anshan birth cohort (MABC) General population 2013–2014	Phthalates (MEHP, MEHHP, MEOHP, MMP, MEP, MBP)	Maternal urine/serum 1st, 2nd, 3rd trimester of pregnancy	*Cognitive development* WPPSI-IV (3–6 years) *Autistic-like behaviors* CHAT (1.5–3 years) CABS (5–6 years)	Prenatal exposure to DEHP and its metabolites associated with an increased risk of ASD-related traits (particularly late-pregnancy exposure). Significant associations at age 3 observed for DEHP, MEHP, MEOHP, and MEHHP. Marginal association for DEHP and MEHP at ages 5–6. *Adjustment confounders* Maternal age, pre-pregnancy BMI, education, ethnicity, household income, smoking during pregnancy, drinking during pregnancy, primipara, lead exposure, and intelligence quotient.	Boys: stronger and earlier associations observed between prenatal phthalate exposure and autistic traits at ages 3 and 6. Girls: similar association, but only at later ages (5–6 years)
Haggerty, et al. (65) US Cohort	*N* = 77 mother-infant dyads (*n* boys = 37; *n* girls = 40) Archives for Research on Child Health (ARCH) Child Development Cohort (ARCH- CDC) General population 2008–2016	Phthalates (MEHP, MEHHP, MEOHP, MECPP, MBP, MiBP, MEP)	Maternal urine 1st trimester of pregnancy	*General behavioral problems* CBCL (3–6 years)—*Autistic-like behaviors* SRS-2 (3–6 years)	No overall association between prenatal phthalates exposure and SRS-2 score; but higher prenatal MEP concentrations associated with higher SRS-2 *T*-scores. *Adjustment confounders* Behavior problems (CBCL).	Boys: positive association between prenatal MEP exposure and SRS-2 scores. Girls: no association observed
Kim et al. (55) South Korea Cohort	*N* = 547 Environment and Development of Children (EDC) General population 2008–2010	Phthalates (MEHHP, MEOHP, MBP, MECPP, MBzP)	Maternal urine 2nd trimester of pregnancy Children urine (4,6,8 years)	Cognitive *development* WISC (6 years) *Autistic-like behaviors* SCQ (4,6,8 years)	Prenatal MEHHP, MEOHP, and MBP exposure associated with higher SCQ scores at age 4, Postnatal MEHHP and MEOHP exposure at age 4 and 8 associated with higher SCQ scores at age 8. *Adjustment confounders* Age, sex (for all children), twin, birth order, maternal education levels, and phthalate levels during pregnancy.	Boys: stronger and significant associations: prenatal and postnatal MEHHP and MEOHP exposure linked to increased SCQ scores in boys at ages 4, 6, and 8 Girls: no significant associations observed *Overall, boys consistently scored higher on SCQ than girls across all ages.*
Miodovnik et al. (56) US Cohort	*N* = 404 Mount Sinai Children's Environmental Health Study General population 1998–2002	Phenols (BPA) Phthalates (MEP, MBP, MiBP, MMP)	Maternal urine 3rd trimester of pregnancy	*Autistic-like behaviors* SRS (7–9 years)	BPA: prenatal exposure to BPA not associated with changes in offspring SRS score at 7–9 years. Phthalates: prenatal exposure to MEP and the sum of LMWP associated with increased changes in children SRS score at 7–9 years. *Adjustment confounders* Maternal age, maternal IQ, marital status at the time of follow-up, maternal education, child race, sex, child IQ, exact age at examination, and urinary creatinine.	No sex-specific effects discussed
Oulhote et al. (51) Canada Cohort	*N* = 556 (*n* boys* = 268; n* girls = 288) Maternal–Infant Research on Environmental Chemicals (MIREC) General population 2008–2011	Phthalates (MBP, MBzP, MEP, MCPP, DEHP)	Maternal urine 1st trimester of pregnancy	*Autistic-like behaviors* SRS-2 (3–4 years)	Maternal exposure to MBP and MCPP associated increased offspring SRS score at 3–4 years. *Adjustment confounders* Child's age at neuropsychological assessment, child sex, birth weight, maternal age at start of pregnancy, maternal education, household income, marital status, mother's country of birth, race/ethnicity, maternal stress index, depressive symptoms, first trimester blood lead concentrations, smoking/alcohol consumption during pregnancy, city and year of enrolment, home caregiving, duration of exclusive breastfeeding.	Boys: positive and stronger association between prenatal MBP exposure and higher SRS scores. Girls: null or negative association: prenatal MEP exposure negatively associated with SRS scores
Patti et al. (60) US Cohort	*N* = 276 mother-infant dyads Health Outcomes and Measures of the Environment (HOME) General population 2003–2006	Phthalates (MCPP, MiBP, MBzP, MBP, DEHP, MEP)	Maternal urine 2nd, 3rd trimester of pregnancy	*Autistic-like behaviors* SRS (4–8 years)	Higher prenatal concentrations of MBP, MBzP, MiBP, MCPP, and DEHP positively associated with increasing child SRS *T*-scores, particularly at higher percentiles. The strongest association observed for MBzP at the 95th percentile. *Adjustment confounders* Maternal age, race, parity, cotinine concentrations, and household income.	Similar associations between boys and girls for MBP, MBzP, MEP, and DEHP at lower SRS percentiles. Stronger associations in boys at the highest percentiles.
Patti et al. (60) US Cohort	*N* = 140 mother-infant dyads Early Autism Risk Longitudinal Investigation (EARLI) High-risk ASD population 2009–2012	Phthalates (MCPP, MiBP MBzP, MBP, DEHP, MEP)	Maternal urine 1st, 2nd, 3rd trimester of pregnancy	*Autistic-like behaviors* SRS (3 years)	Weak or negative association with SRS-*T* scores for MBP, MEP, and DEHP, null for MiBP and MCPP, with only modest increases for MBzP at higher percentiles. *Adjustment confounders* Maternal age, race, parity, cotinine concentrations, and household income.	No sex-specific effect modification observed for MBzP, MiBP, MCPP, or DEHP.
Shin et al. (57) US Cohort	*N* = 201 mother-infant dyads (*n* boys = 122; *n* girls 70) *N* ASD = 46 (*n* boys = 13; *n* girls = 33) Markers of Autism Risks in Babies-Learning Early Signs (MARBLES) High-risk ASD population 2007–2014	Phthalates (MEP, MiBP, MiBP, MBP, MHBP, MBzP, MEHP, MEHHP, MEOHP, MECPP, MCPP, MCOP, MCNP)	Maternal urine 2nd, 3rd trimester of pregnancy	*Cognitive development* MSEL (3 years)- *Autistic-like behaviors* ADOS (3 years)	Null associations of average concentrations of phthalate biomarkers during mid-to late pregnancy with both ASD and non-typical development. MEP only associated with an increased risk of non-typical development in the full sample. *Adjustment confounders* Child's birth year, maternal pre-pregnancy body mass index, homeownership as a proxy of socioeconomic status.	No sex-specific effect observed for phthalates prenatal exposure and increased risk of ASD. Boys: MEP, MBzP, MCPP, MCNP, and DEHP positively associated with non-typical development risk.
Sotelo-Orozco et al. (63) US Cohort	*N* = 148 mother-infant dyads (*n* boys = 93; *n* girls = 55) *N* ASD = 36 Markers of Autism Risks in Babies-Learning Early Signs (MARBLES) High-risk ASD population 2008–2019	Phenols (BPA, BPS, Triclosan, 2,4-dichlorophenol, 2,5-dichlorophenol, Benzophenone-3) Parabens (MEBP, PRPB) Phthalates (MBP, MBzP, MCNP, MCOP, MCPP, MECPP, MECPTP, MEHHP, MEHHTP, MEOHP, MEP, MHBP, MiBP)	Children urine (3–6 months)	*Cognitive development* MSEL (3 years)—*Autistic-like behaviors* ADOS (3 years)	No overall association between children EDCs exposure at 3–6 months and ASD or Non-Typical Development risk. Higher MEP concentrations associated with lower MSEL scores on motor skills and visual perception; higher DEHTP concentrations associated with poorer visual reception and lower composite scores. Higher concentrations of DEHTP, methyl-paraben, MCNP, and BPA associated with lower ASD risk in single-compound analyses. *Adjustment confounders* Child's birth year, the child's sex, maternal age, maternal pre-pregnancy BMI, and parental homeownership	No sex-specific effects discussed
Uldbjerg et al. (62) US Cohort	*N* = 195 children (*n* boys = 108; *n* girls = 87) Preconception Environmental exposures And Childhood health Effects (PEACE) General population 2008-ongoing	Phenols (BPA) Phthalates (MEP, MBP, MiBP, MBzP, MCPP, MCOP, MCNP, DEHP, MEHP, MEHHP, MEOHP, MECPP)	Maternal/paternal preconception urine Maternal urine 1st, 2nd, 3rd trimester of pregnancy	*Autistic-like behaviors* SRS (6–15 years)	No maternal or paternal preconception exposure or maternal prenatal exposure to phthalates or BPA associated with children SRS scores. Inverse, but non-significant associations observed for maternal preconception MiBP and MCOP concentrations and paternal preconception MiBP concentrations, associated with lower SRS *T*-scores. During pregnancy, only MBzP showed a positive but non-significant association with SRS scores. *Adjustment confounders* Parental age, parental educational status, parental body mass index (BMI) pre-pregnancy.	Inverse, non-significant and no clear sex-specific effects observed. Boys: higher maternal preconception levels of MBP, MiBP, MBzP, MCPP, and MCOP and paternal preconception levels of MBP, MiBP, and MBzP tended to be associated with lower SRS scores, while MEP and MCPN showed weak positive associations. Girls: non-significant associations between maternal preconception MBP and MCPP and paternal MiBP levels with higher SRS scores.
Yu et al. (59) China Cohort	*N* = 3,220 (*n* boys = 1,645; *n* girls = 1,575) Ma’anshan birth cohort (MABC) General population 2013–2014	Phthalates (MMP, MEP, MBP, MHWP, MBzP, MEHP, MEOHP, MEHHP)	Maternal urine 1st, 2nd, 3rd trimester of pregnancy	*Autistic-like behaviors* CHAT-A (1.3–5 years) CABS (5–6 years)—*ADHD* and *emotional and behavioral problems* C-ASQ; SDQ (3–6 years)	Higher exposure to MMP, co-exposure to low molecular weight phthalates (MMP, MEP, MBP), and high molecular weight phthalates (MEHP, MEOHP, MEHHP) across pregnancy associated with increased autistic traits. High MBP, MMP, MEP, MBP, MEHP, MEOHP, MEHHP concentrations associated with increased emotional and behavioral problems. MEP and MBzP exposure associated with peer relation problems. *Adjustment confounders* Maternal age, pre-pregnancy body mass index, education, ethnicity, occupation, annual income, primipara, smoking/drinking during gestation, maternal metabolic risk factors	Associations between MMP, LMWP, DEHP, and total phthalate mixture and autistic traits observed in boys but not in girls, although sex was not a significant effect modifier. Boys: conduct problems were associated with MBP and total phthalates; hyperactive behaviors associated with MEP. Girls: emotional problems associated with MMP and MMP, MEP, MBP exposure in girls; hyperactive behaviors associated with MBP.

ADI-R, autism diagnostic interview-revised; ADOS, autism diagnostic observation schedule; ASQ, age & stage questionnaire; BASC-2, behavioral assessment system for children (2nd edition); BP1,2,3,8, bisphenol-1, bisphenol-2, bisphenol-3, bisphenol-8; BPA, bisphenol A; BPAF, bisphenol AF; BPB, bisphenol B; BPF, bisphenol F; BPP, 4,4′-(1,4-phenylenediisopropylidene) bisphenol; BPS, bisphenol S; BPZ, bisphenol Z; BUPB, brominated flame retardants; BuPB, butyl paraben; CHAT, checklist for autism in toddlers; CHAT-A, checklist for autism in toddlers (part A); DDT, dichlorodiphenyltrichloroethane; DEHP, di(2-ethylhexyl) phthalate; ETPB, ethyl paraben; Et-FOSAA, 2-(N-methyl-perfluorooctane sulfonamido) acetate; HCB, hexachlorobenzene; HMW phthalates, high molecular weight phthalate; LMW phthalates, low molecular weight phthalates; MBP, monobutyl phthalate; MBzP, monobenzyl phthalate; M-CHAT, modified checklist for autism in toddlers; MCHHP, mono(2-ethyl-5-hydroxyhexyl) phthalate; MCHP, mono-cyclohexyl phthalate; MCNP, monocarboxyisononyl phthalate; MCOP, monocarboxyoctyl phthalate; MECPP, mono(2-ethyl-5-carboxypentyl) phthalate; MEHHP, mono(2-ethyl-5-hydroxyhexyl) phthalate; MEHP, mono(2-ethylhexyl) phthalate; MEOHP, mono(2-ethyl-5-oxohexyl) phthalate; MEP, mono-ethyl phthalate; MEPB, methyl paraben; Me-FOSAA, 2-(N-ethyl-perfluorooctane sulfonamido) acetate; MIPP, mono-isopentyl phthalate; MMP, mono-methyl phthalate; MNP, monoisononyl phthalate; MOP, mono-n-octyl phthalate; MCPP, mono(3-carboxypropyl) phthalate; MSEL, Mullen scales of early learning; OC, organochlorine; PFAS, perfluoroalkyl substances; PFDA, perfluorodecanoate; PFDoDA, perfluorododecanoate; PFHxS, perfluorohexane sulfonate; PFNA, perfluorononanoate; PFOA, perfluorooctanoate; PFOS, perfluorooctane sulfonate; PFUA, perfluoroundecanoate; PRPB, propyl paraben; SCQ, social communication questionnaire; SDQ, strengths and difficulties questionnaire; SRS, social responsiveness scale; TCC, triclocarban; TCS, triclosan; WPPSI-IV, Wechsler preschool and primary scale for intelligence (4th edition); WISC, Wechsler intelligence scale for children.

### Effects of prenatal exposure to phthalates

Across the included studies, the metabolites most consistently associated with ASD-related outcomes included MBP, MCPP, MBzP, and several DEHP metabolites, particularly MEHHP and MEOHP. Evidence from the Canadian Maternal–Infant Research on Environmental Chemicals (MIREC) cohort supports an association between first-trimester phthalate exposure and autistic traits in early childhood (ages 3–4 years). Using data from the same cohort, Oulhote et al. (69) and Alampi et al. (70) independently reported that higher maternal urinary concentrations of MBP and MCPP during early pregnancy were associated with higher total SRS scores at 3–4 years of ages, indicative of greater social difficulties. Oulhote et al. (69) also reported associations across several SRS domains, including social cognition, social communication, social motivation, and restricted and repetitive behaviours.

Similar findings were observed in other prospective cohorts. In the TIDES cohort, Day et al. (71) reported that higher cumulative exposure to prenatal phthalate mixtures during early pregnancy was associated with increased autistic traits at ages 4 and 5 years, with MCPP, MBzP, and MEP contributing most strongly to the overall mixture effect. Likewise, Kim et al. (72), using data from the Korean Environment and Development of Children (EDC) cohort, found that second-trimester exposure to DEHP-related metabolites, particularly MEHHP and MEOHP, was associated with higher SCQ scores at 4 years of age. In the Mount Sinai Children's Environmental Health Study, Miodovnik et al. (73) similarly reported that higher third-trimester concentrations of low-molecular-weight phthalates metabolites were associated with greater autistic traits, with MEP showing the strongest association.

Other studies highlighted the importance of considering exposure across the entire pregnancy. Using data from the Ma'anshan Birth Cohort (MABC), Gao et al. (74) found that prenatal exposure to DEHP and its metabolites, was associated with an increased risk of ASD-related traits in early childhood, at approximately 3 years of age. Similarly, Yu et al. (75) characterised longitudinal exposure throughout pregnancy using data from Mount Sinai Children's Environmental Health Study. The authors found that higher exposure to MMP, low-molecular-weight phthalates, DEHP metabolites, and total phthalate mixtures was associated with an increased probability of autistic traits. Together, these findings suggest that both cumulative prenatal exposure and exposure during specific developmental windows may contribute to ASD-related outcomes.

More recent studies have supported these observations, although with substantial between-cohort variability. In the HOME cohort, Patti et al. (76) reported positive associations between prenatal exposure to phthalates metabolites, including MBP, MiBP, MBzP, MCPP, and DEHP, and higher SRS scores, with the strongest associations observed for MBzP. Emma et al. (77) reported a similar pattern, although the associations did not reach statistical significance. In contrast, analyses from the high-risk EARLI cohort showed weaker and non-significant associations for the same metabolites, suggesting that the relationship between prenatal phthalate exposure and autistic traits may differ according to population characteristics or underlying ASD risk.

Not all studies reported significative findings. In the MARBLES cohort, Shin et al. (78) found no significant associations between maternal phthalate concentrations during the second and third trimesters and neurodevelopmental outcomes, except for MEP, which was associated with an increased risk of non-typical development rather than ASD. Similarly, Uldbjerg et al. (79), using data from the PEACE cohort, found no significant associations between prenatal phthalate exposure and children's SRS scores, although weak positive associations were observed for prenatal MBzP. Al-Saleh et al. (80), in the Saudi Early Autism and Environment Study, also reported limited evidence, with an association observed only between higher third-trimester MiBP concentrations and lower personal-social functioning on the ASQ-3.

### Effects of post-natal exposure to phthalates

In the Korean Environment and Development of Children (EDC) cohort, Kim et al. (72) reported that higher urinary concentrations of the DEHP metabolites MEHHP and MEOHP at 4 and 8 years of age were associated with higher SCQ scores at age 8, suggesting that ongoing childhood exposure may contribute to autistic traits beyond the prenatal period.

In contrast, findings from the MARBLES cohort did not support a consistent association between early postnatal phthalate exposure and ASD risk. Sotelo-Orozco et al. (81) observed no overall association between phthalate concentrations measured during the first months of life and either ASD or non-typical development. Although higher MEP concentrations were associated with lower motor and visual-perception scores, and other metabolites, such as monocarboxyisononyl phthalate (MCNP) were inversely associated with ASD risk in single-compound analyses, these findings were not consistent across neurodevelopmental outcomes.

Evidence from case-control studies also remains limited. In the CHARGE study, Bennett et al. (82) found that MCINP was the only individual metabolite significantly associated with increased odds of ASD, while mixture analyses suggested that phthalates mixture was associated with higher odds of both ASD and developmental delay. MCPP and MEHHP were found among the principal contributors to the mixture effect.

### Early-life phthalate exposure and sex-specific vulnerability to ASD

A limited number of studies have explored whether the association between early-life phthalate exposure and ASD-related outcomes differs between males and females.

The strongest evidence suggesting greater male susceptibility comes from the Canadian MIREC cohort. Using data from the same population, Oulhote et al. (69) and Alampi et al. (70) consistently reported stronger associations between first-trimester exposure to phthalate metabolites, particularly MBP, MEP, and MBzP, and higher SRS scores among boys than girls. Oulhote et al. (69), further showed that higher prenatal MBP exposure was associated with greater difficulties across multiple domains of social functioning in boys, whereas comparable associations were not observed among girls. Similar trends were reported in the Ma'anshan Birth Cohort. Gao et al. (74) observed that prenatal phthalate exposure was associated with ASD-related traits earlier and more consistently in boys (age 3), whereas comparable patterns in girls emerged only at later ages (age 5–6). Likewise, Yu et al. (75) found that prenatal exposure to MMP was associated with autistic traits among boys but not among girls. Although the authors also reported sex differences in emotional, hyperactive, and conduct problems, formal interaction analyses provided only limited evidence of effect modification by sex (75). Evidence of greater susceptibility among boys was also reported by Haggerty et al. (83), who found that higher prenatal MEP concentrations were associated with increased SRS scores among boys only. However, the absence of statistically significant interaction effects limits the strength of conclusions on true sex-specific vulnerability.

Evidence suggesting greater susceptibility among girls was more limited. Al-Saleh et al. (80) reported that first-trimester MiBP exposure was associated with lower personal-social development among girls but not boys. However, this finding has not been consistently replicated in other cohorts.

Other studies included in this review reported inconsistent or unclear findings that did not support meaningful sex differences. In the HOME cohort, both Patti et al. (76) and Emma et al. (77) reported similar associations between prenatal phthalate exposure and SRS scores in boys and girls. Likewise, Shin et al. (78) found no statistically significant interaction between prenatal phthalate exposure and child sex, despite observing positive associations between several phthalates (MEP, MBzP, MCPP, MCNP, DEHP) and non-typical development risk in boys. Similarly, Uldbjerg et al. (79) reported inconsistent and non-significant sex-specific patterns across maternal and paternal preconception exposures, with no clear evidence supporting differential sex susceptibility. Finally, Bennett et al. (82), Choi et al. (84), Miodovnik et al. (73), Sotelo-Orozco et al. (81), Barosky et al. (85), and Braun et al. (86) did not report sex-stratified analyses or sex-specific effect modification.

## Discussion

The aim of this systematic review was to synthesise the current evidence on sex-specific susceptibility to phthalates exposure and the association between such exposure and the risk of developing autism spectrum disorder (ASD). Overall, most epidemiological studies suggest that prenatal exposure to phthalates is associated with higher levels of autistic traits and ASD-related behaviours, although findings remain heterogeneous across cohorts, exposure windows, and metabolites. Positive associations have been reported in different prospective birth cohorts, while a smaller number of studies found null associations. It is important to emphasise the considerable heterogeneity among the reviewed studies in terms of participants characteristics and methodological approaches, as well as the type and number of phthalates metabolites investigated. Thus, the frequency with which a specific metabolite was found to be associated with ASD in the present review may partly reflect differences in metabolites selection across studies. With this in mind, we identified a recurring pattern of associations for specific phthalate metabolites, particularly MBP, MEP, MBzP, MCPP and DEHP-related metabolites including MEHP, MEHHP, MEOHP, MECPP.

The most consistent associations were observed for MBP and MCPP, particularly during early pregnancy (68–70). During late pregnancy, associations appear more heterogeneous but remain evident for DEHP and its metabolites (MEHP, MEHHP, MEOHP) (71–74) and for LMW phthalates, especially MEP (72). When findings were integrated across studies, MBP, MEP, MBzP, and DEHP metabolites emerged as the most frequently associated with ASD-related outcomes. However, associations between phthalates metabolites and ASD-related outcomes varied according to cohort design and exposure window. For example, analyses from the HOME cohort (75, 76) showed generally positive associations for MBP, MBzP, and DEHP-related mixtures, although these were not always statistically robust, whereas the high-risk EARLI cohort (77), showed weaker or null associations.

Evidence regarding postnatal exposure remains limited because few of the included studies examined this exposure window. Only two studies addressed this developmental window. Kim et al. (71) reported that postnatal concentrations of MEHHP and MEOHP were associated with higher SCQ scores at age 8, while Sotelo-Orozco et al. (80) reported largely null or inverse associations depending on the compound, suggesting that associations with postnatal may be weaker or less consistent.

Autism spectrum disorder is consistently reported to be more prevalent in males than in females, with a male-to-female ratio of approximately 3–4:1 (4, 6, 87). Although this sex bias is well established across epidemiological studies, its underlying mechanisms remain incompletely understood. Recent reviews suggest that this sex bias may reflect a combination of sex-specific genetic architecture, differential brain gene expression, hormonal influences, and possible under-recognition of female ASD presentations (88). Large population-based studies indicate that ASD liability is strongly influenced by inherited genetic factors, with heritability estimates around 80% (89). However, this substantial genetic contribution does not exclude the potential role of non-genetic exposures occurring during sensitive developmental windows. Accordingly, increasing attention has focused on non-genetic factors that may contribute to ASD risk, including prenatal exposure to environmental chemicals and its possible association with ASD-related traits (7, 8).

Overall, the available evidence suggests that boys may be more susceptible to the neurodevelopmental effects of prenatal phthalate exposure. However, findings remain inconsistent across cohorts, and statistically significant effect of modification by sex has not been consistently demonstrated across the included studies. Based on the limited and statistically weak evidence that we were able to gather, boys may be more vulnerable to certain phthalates metabolites such as MBP, MEP, and DEHP-related metabolites. The MIREC cohort showed stronger and more consistent associations in boys across all SRS domains, particularly in the upper percentiles of autistic trait distribution, whereas no significant associations were observed in girls. Results from the MABC cohort further supported this pattern, showing earlier and stronger exposure–response relationships among boys, whereas similar associations among girls emerged later in development. Other cohorts (e.g., ARCH-CDC, MARBLES) provided partial support for a male vulnerability. For example, Haggerty et al. (83) observed associations between MEP and ASD traits only among boys, while Shin et al. (78) reported non-significant but male-driven associations with non-typical development in mixture models rather than ASD traits.

Studies that used additional neurodevelopmental and behavioural assessment tools alongside autism-specific instruments, reported findings suggestive of female-specific susceptibility. For example, Al-Saleh et al. (80) found that first-trimester exposure to MiBP was negatively associated with all ASQ-3 subscales in girls, with a particularly pronounced effect on the personal–social domain. Although ASQ-3 is not a direct measure of autistic-like behaviours, lower scores on its personal–social functioning domain may reflect early difficulties in social communication and thus may represent potential early indicators of ASD-related traits. Similarly, Yu et al. (75), using the Strengths and Difficulties Questionnaire (SDQ) and the Conners Abbreviated Symptom Questionnaire (C-ASQ), observed that certain phthalates (MBP, MEP, MMP) were associated with emotional problems among girls but not among boys. In contrast, several cohort studies (HOME, EARLI, PEACE, CHARGE) reported no clear sex-specific association or showed inconsistent patterns (75, 76, 78, 81).

The neurodevelopmental effects of phthalates are usually discussed within a neurotoxicological framework, whereby phthalates and their metabolites disrupt brain homeostasis during critical periods of development. Phthalate metabolites can cross the placenta and have been detected in placental tissues, cord blood, amniotic fluid with a strong potential for disrupting normal embryonic and foetal development (90). The hypothesised mechanisms are numerous and complex, and include disruption of endocrine homeostasis, oxidative stress, inflammation and epigenetic reprogramming of the placenta and brain (10, 11). For example, higher concentration of phthalates in maternal urines during the first trimester have been associated with altered methylation of placental genes (91). Additionally, phthalates may disrupt endocrine signalling by altering hormone binding to and the transcriptional activity of sex-steroids and thyroid hormone receptors (92). Higher concentration of phthalate metabolites during pregnancy have been associated with lower thyroid hormones concentration (93–95) and maternal hypothyroxinaemia, especially in early pregnancy, has been associated with a higher risk for ASD in the offspring, although findings are heterogenous (96, 97), and with lower androgen production in infants (50, 98–101). Phthalates and their metabolites may also affect mitochondrial functioning, microglia activation and astrocyte stress response, processes that are generally linked to neuroinflammation (102). Disruption of these processes can affect neurogenesis and proliferation, synaptic density and function with potential consequences for cognition and behaviour (52–55, 103).

Presumably, within the same cohort boys and girls are exposed to similar levels of phthalates. So how then might males may be at higher risk for ASD following exposure to certain phthalates? One possibility is that boys and girls show different biological susceptibility to environmental stressors. On the one hand, females may require a higher mutational, and perhaps environmental, burden than males to meet the diagnostic threshold for ASD, suggesting greater resilience among females (18, 19, 104). On the other hand, males may be more vulnerable because of the processes underlying the developmental masculinization of the brain (21–23, 25, 26).

Based on the observed positive correlation between foetal oestrogens and androgens concentrations with ASD risk, the sex steroid hypothesis of ASD (21) proposes that factors that increase sex-steroids production or affect genetic networks involved in brain masculinization may increase the likelihood of being diagnosed with ASD. During development, sex steroids influence gene regulation in nervous system cells, neurogenesis, cell proliferation and apoptosis, the balance between excitatory and inhibitory neuronal subtypes (26, 27) and neuroimmune response processes (105). In this regard, phthalates may play an important role because of their complex effects on the placenta and on the foetal testes, two organs involved in steroidogenesis during gestation (106, 107). Animal studies demonstrate that the cellular and the molecular actions of phthalates are complex and depend on the species or strain examined, the timing of exposure and the specific phthalate or metabolite investigated (108). These complex actions of phthalates do not yet allow a well-informed and predictive model of their sex-specific effects. However, phthalates may interfere with systems that are clearly involved in brain sexual differentiation, making them plausible contributors to ASD risk, especially among boys and when exposure occurs early in development.

Although the reviewed literature lends some support to the hypothesis of a sex-dependent susceptibility to the effects of early phthalates exposure, it is important to stress that this hypothesis has not been tested sufficiently rigorously, and the conclusion should be interpreted with caution. Several factors may explain the lack of consistent sex-specific effects in the literature reviewed. One explanation is that most of the studies were not designed to test a robust effect on sex differences as were often underpowered to detect statistically significant interaction term. Other explanations related to the heterogeneity of the methods used by the different studies: the group of phthalates and metabolites investigated varies across studies as well as the time window of exposure. In our selection of articles, we tried to reduce the heterogeneity of the methods used to measure ASD outcome. Nonetheless, variation across studies in the assessment instrument used and the ages at assessment likely contributed meaningfully to the heterogeneity of results. These limitations have also been discussed by others (55, 57). Another explanation concerns the tests instruments used to assess autistic traits. It is possible that in the way in which these instruments were designed and validated makes them more sensitive to detect symptoms in males (15, 109–111). Lastly, few studies reported sex-specific scores for the subdomains of the different assessment instruments.

Reporting that scores is important both for statistical good practices but also for theoretical reasons. The “extreme male brain” hypothesis proposes that autism traits represent an extreme of a predominantly male-typical cognition (112, 113). This hypothesis has its foundations on the reported sex-differences on two dimensions of psychological traits: empathizing, the ability to understand others' feelings and thoughts, and systemizing, the preferences for identifying patterns of relationship and rules in non-social domains (25). These traits are normally distributed in the general population, with females generally scoring higher than males in the empathizing domain and males generally scoring higher than females on systemizing. Males and females autism diagnosis tend to score towards the extreme male-typical end of the distribution for these traits (114). Higher empathizing scores among females reflect their higher performance on tasks related to the social domains, such as communication, emotion recognition and theory of mind, with these differences becoming larger when these measures are combined (115). Describing whether and how the males and females differ across domains and sub-domains of the autism spectrum, in addition to developing instruments that are more sensitive to autism in females, would provide important information necessary for understanding sex-specific vulnerability.

In conclusion, to test the hypothesis of sex-differences to environmental exposure, particularly phthalates, in relation to ASD, future studies should be sufficiently powered to conduct sex-stratified analyses and detect interaction terms; they should use harmonised measures, in conjunction with non- clinical measures designed to detect sex differences and consider timing and intensity of exposure more systematically.

## Limitations

Several limitations should be considered. First, because the review was specifically focused on ASD, this inclusion criteria may have reduced the number of eligible studies. For example, studies investigating broader neurodevelopmental or behavioural outcomes were excluded, with the possibility that these may include relevant subdomains related to core ASD features (e.g., social communication and interaction, or personal–social functioning). Second, although the initial title and abstract screening, full-text assessment, and risk-of-bias evaluation were not conducted independently by two reviewers, eligibility decisions for the final set of potentially relevant full-text articles were discussed with a second reviewer, and consensus was reached regarding the final set of included studies. The risk-of-bias assessment was also reviewed and discussed with a second reviewer to improve consistency of judgement. Therefore, although the lack of a fully independent duplicate screening and appraisal process represents a methodological limitation, it was partially mitigated through consensus-based discussion throughout the review process, including study selection and quality assessment.

Third, a recurrent limitation among the included studies concerned ASD assessment: most relied on parent-reported screening instruments, particularly the SRS, rather than clinical diagnostic assessments of ASD. Although SRS is strongly correlated with gold-standard diagnostic tools such as the ADI-R and the ADOS, it is not diagnostic and may be subject to parent-report bias (116, 117). Moreover, SRS scores may be significantly influenced by cognitive ability and general behavioural problems, meaning that higher scores may not always reflect core ASD symptoms but rather broader developmental or behavioural difficulties (118). Finally, across the included literature emerged the problem of results generalisability, as several cohorts were characterised by relatively homogeneous populations, often with higher socioeconomic status, higher educational background, or specific risk profiles (e.g., high familial risk cohort) potentially limiting the external validity of the findings.

## Data Availability

The original contributions presented in the study are included in the article/Supplementary Material, further inquiries can be directed to the corresponding author.
