# Supplementary material for: Sex-dependent vulnerability to early-life phthalates exposure and autism spectrum disorders: a systematic review
Source: Front Child Adolesc Psychiatry. 2026 Aug 11;5:1865057. doi: 10.3389/frcha.2026.1865057 (PMC13503302; doi:10.3389/frcha.2026.1865057)
Supplement: Supplementary file 1 [file Table1.docx]

| **Table 1. Summary of the characteristics of included studies examining environmental chemicals exposure and ASD outcomes, including sex specific effects.** | | | | | | |
| --- | --- | --- | --- | --- | --- | --- |
| **Author, year, country, study design** | **Sample size, population and period of study** | **Type of EDC** | **Biological matrix and timing of exposure** | **Outcomes, assessment tools and age at assessment** | **Main findings** | **Sex-specific effects** |
| Alampi et al. (52)  Canada  Cohort | N = 478 mother–infant dyads  (*n* boys=235; *n* girls =243)  Maternal–Infant Research on Environmental Chemicals (MIREC)  General population  2008-2011 | **Phenols** (BPA)  **Phthalates** (MBP, MBzP, MCPP, MEHHP, MEHP, MEOHP, MEP)  **Metals** (arsenic, cadmium, lead, manganese, mercury) | Maternal blood/plasma/urine  1^st^ trimester of pregnancy (6–13 weeks’ gestation) | *Autistic-like behaviors*  SRS-2  (3-4 years) | Higher levels of lead, cadmium, bisphenol-A, mono-3-carboxypropylphthalate (MCPP), mono-butyl phthalate (MPB) associated with higher mean SRS scores.  *Adjustment confounders*  Child sex, folic acid supplementation, caregiver environment score, household income, relationship status, maternal ethnicity, age, education, parity, and city of residence. | Boys: stronger associations for MBP, MEP, and MBzP  Girls: stronger associations for oxychlordane and trans-nonachlor.  *Differences in associations between the sexes were pronounced at the 90th percentile of SRS scores.* |
| Al-Saleh et al. (58)  Saudi Arabia  Cohort | N = 456 children  (*n* boys=334; *n* girls =*121*)  N children (full assessment) = 125  N ASD=51  (*n* ASD= 30; *n* girls ASD=20  Saudi Early Autism and Environment (SEAES)  General population  2019-2022 | **Phenols** (BPA)  **Phthalates** (MEP, MiBP, MBP, MBzP, MECPP, MEHHP, MEOHP) | Maternal urine  1^st^, 2^nd^, 3^rd^ trimester of pregnancy | *Neurodevelopment*  ASQ-3  (6, 12,18 months)  -  *Autistic-like behaviors*  M-CHAT  (18 months) | Associations between ASQ-3 scores and phthalate metabolites/BPA observed across the three trimesters, mainly in communication, gross motor, and problem-solving domains.  Higher MBP, MiBP, and BPA 1^st^ trimester exposure linked to decreased gross motor scores.  MiBP 3^rd^ trimester exposure and BPA 2^nd^ trimester exposure associated with lower personal-social scores.  MEP and low-molecular-weight phthalates linked to improved communication scores.  Some 1^st^ trimester exposures (MEP, MEOHP) associated with lower odds of a positive M-CHAT screen, while 1^st^ trimester MBP exposure associated with potential increase in risk.  *Adjustment confounders*  Maternal weight gain, gravidity, education level, health conditions, medications, supplements, herbal remedies, pregnancies assisted by IVF or infertility drugs, exposure to environmental tobacco smoke, and the infant’s age and gender. | Boys: stronger adverse associations in problem-solving and social domains.  Girls: more affected in gross and fine motor scores.  2^nd^ trimester BPA exposure associated with lower personal-social scores in both sexes  1^st^ trimester MiBP exposure affected personal-social scores only in girls.  *ASQ is not a direct measure of autistic like behaviors, but lower score in personal social domain may indicate potential autism-like behaviors.* |
|  |  |  |  |  |  | (*Continued*) |
| Barkoski et al. (68)  US  Cohort | N= 207 children  (*n* boys =122; *n* girls=85)  N ASD=47  (*n* ASD= 31; *n* girls ASD=16  Markers of Autism Risks in Babies-Learning Early Signs (MARBLES)  High-risk ASD population  2007-2014 | **Phenols** (BPA, BPS, BPF, TCC, TCS)  Parabens (BuPB, ETPB, MEPB, PRPB) | Maternal urine  2^nd^, 3^rd^ trimester of pregnancy | *Cognitive development*  MSEL  (3 years)  -  *Autistic-like behaviors*  ADOS  (3 years) | Prenatal exposure to BPA and parabens not associated with the risk of ASD at 3 years old.  *Adjustment confounders*  Pre-pregnancy body mass index (BMI), peri-conceptional prenatal vitamin use, homeowner status, birth year, and child's sex. | No sex-specific effects observed. |
| Bennett et al. (64)  US  Case control | N= 627 children  (*n* boys=484; *n* girls =143)  N ASD = 224  (*n* boys=181; *n* girls =43)  Childhood Autism  Risks from Genetics and Environment (CHARGE)  General population and ASD  2006-2017 | **Phenols** (BP1,2,3,8, BPA, BPF, BPAF, BPB, BPF, BPP, BPS, BPZ, BUPB, DCP24,25, DHB34, ETPB, HP4, HEPB, OH4BP, OHETP, OHMEP, PCP, TCC, TCP245,246, TCS)  **Phthalates** (MiBP, MCHP, MCHPP, MCINP, MCIOP, MCHHP, MECPP, MEHHP, MEOHP, MEP, MHXP, , MINP, MIPP, MMP, MBP, MOP)  **Pesticides** (DMP, DEP, DEMTP, DETP, DMDP, DEDP)  **Trace Elements** (As, Be, Cd, Mo, Tl, U) | Children urine  2-5 years | *Cognitive development*  MSEL  (2-5 years)  -  *Autistic-like behaviors*  ADI-R  (2-5 y)  ADOS  (2-5 y)  -  *Adaptive behavior*  (2-5 y) | Phenols/Parabens: higher children’s urinary concentrations of MEPB, DHB34, and PRBP were associated with increased odds of ASD. Mixture analyses: DEP, MEPB, PRPB, ETPB, and MCINP contributed to ASD risk, while MEPB, TCP246, DHB34, MCPP, and MEHHP contributed to developmental delay risk.  Phthalates: Only higher MCINP levels associated with increased odds of ASD.  Mixture effect analyses: positive associations with both ASD and developmental delay.  *Adjustment confounders*  Child’s sex, year of birth, age in months at time of diagnosis, and race, as well as parental homeowner status, and maternal metabolic conditions during pregnancy. | No sex-specific effects discussed. |
| Braun et al. (69)  US  Cohort | N = 175 mother-infant dyads  (*n* boys =80; *n* girls = 95)  Health Outcomes and Measures of the Environment (HOME)  2003-2006 | **Phenols** (BPA)  **Phthalates** (MEP, MiBP, MBzP, MCPP, MEHP, MEHHP, MECPP)  PFAS (PFOA, PFOS, PFNA, PFHxS)  **OC pesticides** (trans-nonachlor, DDT, HCB) | Maternal urine/blood  1^st^, 2^nd^, 3^rd^ trimester of pregnancy | *Autistic-like behaviors*  SRS  (4-5 years) | Phthalates, BPA: No association with SRS scores at 4–5 years.  Trans-nonachlor and p’p’-DDT: Higher maternal exposure associated with higher SRS scores.  *Adjustment confounders*  Maternal age at delivery, race, marital status, education, parity, insurance status, employment, household income, prenatal vitamin use, depressive symptoms, maternal IQ, tobacco smoke exposure, caregiving environment. | No overall sex differences found for phthalates, BPA, and BFR exposures.  Girls: maternal exposure to HCB and trans-nonachlor was associated with higher SRS score. |
| Choi et al. (66)  US  Cohort | N = 110 mother-infant dyads  (*n* boys =69; *n* girls 41)  N ASD = 25  (*n* boys = 19; *n* girls =6)  Markers of Autism Risks in Babies-Learning Early Signs (MARBLES)  High-risk ASD population  2006-2015 | **Phenols** (BPA, BPS, BPF, TCC, TCS)  **Phthalates** (MEP,  MiBP, MBP, MHBP, MBzP, MEHP, MEHPP, MEOHP, MECPP, DEHP, MCPP, MNP, MCOP, MCNP)  **PFAS** (PFHxS, PFOA, PFOS, PFNA, PFDA, PFUA, PFDoDA, Me-FOSAA, Et-FOSAA) | Maternal urine/blood  1^st^, 2^nd^, 3^rd^ trimester of pregnancy | *Cognitive development*  MSEL  (3 years)  -  *Autistic-like behaviors*  ADOS  (3 years) | Prenatal exposure to PFAS, parabens/triclosan and phthalates/BPA associated with increased ASD risk.  *Adjustment confounders*  Child sex, year of birth, maternal age, maternal race/ethnicity, parity, maternal pre-pregnancy BMI, maternal education, and homeownership. | No sex-specific effects discussed.  (*Continued*) |
| Day et al. (54)  US  Cohort | N = 501 mother-infant dyads  The Infant Development and Environment Study (TIDES)  General population  2010-2012 | **Phthalates** (MEP, MBP, MiBP, MBzP, MEHP, MEOHP, MEHHP, MECPP, MCPP) | Maternal urine  1^st^ and 3^rd^ trimester of pregnancy  (6-21; 24-42 week’s gestation) | *Behavioral problems*  BACS-2  (4-6 y)  *Autistic like behaviors*  SRS-2  (4-6 years) | Early prenatal exposure to phthalates (MCPP, MBzP, MEP) associated with increased autistic traits in both sexes.  Weaker associations with behavioral and adaptive outcomes.  *Adjustment confounders*  Pre-pregnancy BMI, study centre, race, income, maternal education, child sex, parity, time of the day and gestational age at urine collection, self-reported alcohol and cigarette consumption during pregnancy, and maternal depression and stress. | Boys: late prenatal phthalates exposure (3^rd^ trimester) associated with higher externalizing behaviors scores (BACS-2)  Girls: early prenatal phthalates exposure (1^st^ trimester) associated with worse adaptive skill scores (BACS-2) |
| Emma et al. (61)  US  Cohort | N = 271 mother-infant dyads  Health Outcomes and Measures of the Environment (HOME)  General population  2003-2006 | **Phthalates** (MBP, MBzP, MECPP, DEHP, MEP, MiBP) | Maternal urine  2^nd^, 3^rd^ trimester of pregnancy | *Autistic-like behaviors*  SRS  (4-8 years) | Mixture analysis: prenatal phthalate exposure *positive but non-significant* associated with SRS scores.  Higher prenatal exposure to MBP, MBzP, MiBP, MCPP, and DEHP associated whit increasing SRS T-scores, particularly at higher percentiles.  *Adjustment confounders*  Maternal age at delivery, maternal race/ethnicity, maternal parity, pre-pregnancy BMI, household annual income child sex at birth child age at SRS administration, and study site. | Mixture analysis: positive but non-significant associations between prenatal phthalates exposure and SRS score both in males and females.  Similar associations for MBP, MBzP, MEP, and DEHP between boys and girls at lower SRS percentiles; stronger associations in boys at the highest percentiles |
| Emma et al. (61)  US  Cohort | N = 166 mother-infant dyads  Early Autism Risk Longitudinal Investigation (EARLI)  High-risk ASD population  2009-2012 | **Phthalates** (MBP, MBzP, MECPP, DEHP, MEP, MiBP) | Maternal urine  1^st^, 2^nd^, 3^rd^ trimester of pregnancy | *Autistic-like behaviors*  SRS  (3 years) | Mixture analysis: prenatal phthalate exposure *negative but non-significant* associated with SRS scores.  Weak or negative associations or negative for MBP, MEP, and DEHP, and null for MiBP and MCPP across SRS percentiles.  *Adjustment confounders*  Maternal age at delivery, maternal race/ethnicity, maternal parity, pre-pregnancy BMI, household annual income child sex at birth child age at SRS administration, and study site. | Mixture analysis: negative but non-significant associations between prenatal phthalates exposure and SRS score both in males and females.  No effect modification by child sex observed for MBzP, MiBP, MCPP, or DEHP. |
| Gao et al. (53)  CHINA  Cohort | N= 3209 mother-infant dyads  (*n* boys=1639; *n* girls=1570)  Ma'anshan birth cohort (MABC)  General population  2013-2014 | **Phthalates** (MEHP, MEHHP, MEOHP, MMP, MEP, MBP) | Maternal urine/serum  1^st^, 2^nd^, 3^rd^ trimester of pregnancy | *Cognitive development*  WPPSI-IV  (3-6 years)  *Autistic-like behaviors*  CHAT  (1.5-3 years)  CABS  (5-6 years) | Prenatal exposure to DEHP and its metabolites associated with an increased risk of ASD-related traits (particularly late-pregnancy exposure).  Significant associations at age 3 observed for DEHP, MEHP, MEOHP, and MEHHP.  Marginal association for DEHP and MEHP at ages 5–6.  *Adjustment confounders*  Maternal age, pre-pregnancy BMI, education, ethnicity, household income, smoking during pregnancy, drinking during pregnancy, primipara, lead exposure, and intelligence quotient. | Boys: stronger and earlier associations observed between prenatal phthalate exposure and autistic traits at ages 3 and 6.  Girls: similar association, but only at later ages (5–6 years) |
|  |  |  |  |  |  | (*Continued*) |
| Haggerty, et al. (65)  US  Cohort | N =77 mother-infant dyads  (*n* boys =37; *n* girls = 40)  Archives for Research on Child  Health (ARCH) Child Development Cohort (ARCH- CDC)  General population  2008-2016 | **Phthalates** (MEHP, MEHHP, MEOHP, MECPP, MBP, MiBP, MEP) | Maternal urine  1^st^ trimester of pregnancy | *General behavioral problems*  CBCL  (3-6 years)  -  *Autistic-like behaviors*  SRS-2  (3-6 years) | No overall association between prenatal phthalates exposure and SRS-2 score; but higher prenatal MEP concentrations associated with higher SRS-2 T-scores.  *Adjustment confounders*  Behavior problems (CBCL). | Boys: positive association between prenatal MEP exposure and SRS-2 scores.  Girls: no association observed |
| Kim et al. (55)  South Korea  Cohort | N = 547  Environment and Development of Children (EDC)  General population  2008-2010 | **Phthalates** (MEHHP, MEOHP, MBP, MECPP, MBzP) | Maternal urine  2^nd^ trimester of pregnancy  Children urine  (4,6,8 years) | Cognitive *development*  WISC  (6 years)  *Autistic-like behaviors*  SCQ  (4,6,8 years) | Prenatal MEHHP, MEOHP, and MBP exposure associated with higher SCQ scores at age 4, Postnatal MEHHP and MEOHP exposure at age 4 and 8 associated with higher SCQ scores at age 8.  *Adjustment confounders*  Age, sex (for all children), twin, birth order, maternal education levels, and phthalate levels during pregnancy. | Boys: stronger and significant associations: prenatal and postnatal MEHHP and MEOHP exposure linked to increased SCQ scores in boys at ages 4, 6, and 8  Girls: no significant associations observed  *Overall, boys consistently scored higher on SCQ than girls across all ages.* |
| Miodovnik et al. (56)  US  Cohort | N = 404  Mount Sinai Children's Environmental Health Study  General population  1998-2002 | **Phenols** (BPA)  Phthalates (MEP, MBP, MiBP, MMP) | Maternal urine  3^rd^ trimester of pregnancy | *Autistic-like behaviors*  SRS  (7-9 years) | BPA: prenatal exposure to BPA not associated with changes in offspring SRS score at 7-9 y.  Phthalates: prenatal exposure to MEP and the sum of LMWP associated with increased changes in children SRS score at 7-9 y.  *Adjustment confounders*  Maternal age, maternal IQ, marital status at the time of follow-up, maternal education, child race, sex, child IQ, exact age at examination, and urinary creatinine. | No sex-specific effects discussed |
| Oulhote et al. (51)  CANADA  Cohort | N = 556  (*n* boys *= 268; n* girls = 288)  Maternal–Infant  Research on  Environmental  Chemicals (MIREC)  General population  2008-2011 | **Phthalates** (MBP, MBzP, MEP, MCPP, DEHP) | Maternal urine  1^st^ trimester of pregnancy | *Autistic-like behaviors*  SRS-2  (3-4 years) | Maternal exposure to MBP and MCPP associated increased offspring SRS score at 3-4 y.  *Adjustment confounders*  Child’s age at neuropsychological assessment, child sex, birth weight, maternal age at start of pregnancy, maternal education, household income, marital status, mother’s country of birth, race/ethnicity, maternal stress index, depressive symptoms, first trimester blood lead concentrations, smoking/alcohol consumption during pregnancy, city and year of enrolment, home caregiving, duration of exclusive breastfeeding. | Boys: positive and stronger association between prenatal MBP exposure and higher SRS scores.  Girls: null or negative association: prenatal MEP exposure negatively associated with SRS scores |
|  |  |  |  |  |  | (*Continued*) |
| Patti et al. (60)  US  Cohort | N = 276 mother-infant dyads  Health Outcomes and Measures of the Environment (HOME)  General population  2003-2006 | **Phthalates** (MCPP, MiBP  MBzP, MBP, DEHP, MEP) | Maternal urine  2^nd^, 3^rd^ trimester of pregnancy | *Autistic-like behaviors*  SRS  (4-8 years) | Higher prenatal concentrations of MBP, MBzP, MiBP, MCPP, and DEHP positively associated with increasing child SRS T-scores, particularly at higher percentiles.  The strongest association observed for MBzP at the 95th percentile.  *Adjustment confounders*  Maternal age, race, parity, cotinine concentrations, and household income. | Similar associations between boys and girls for MBP, MBzP, MEP, and DEHP at lower SRS percentiles.  Stronger associations in boys at the highest percentiles. |
| Patti et al. (60)  US  Cohort | N = 140 mother-infant dyads  Early Autism Risk Longitudinal Investigation (EARLI)  High-risk ASD population  2009-2012 | **Phthalates** (MCPP, MiBP  MBzP, MBP, DEHP, MEP) | Maternal urine  1^st^, 2^nd^, 3^rd^ trimester of pregnancy | *Autistic-like behaviors*  SRS  (3 years) | Weak or negative association with SRS-T scores for MBP, MEP, and DEHP, null for MiBP and MCPP, with only modest increases for MBzP at higher percentiles.  *Adjustment confounders*  Maternal age, race, parity, cotinine concentrations, and household income. | No sex-specific effect modification observed for MBzP, MiBP, MCPP, or DEHP. |
| Shin et al. (57)  US  Cohort | N = 201 mother-infant dyads  (*n* boys =122; *n* girls 70)  N ASD = 46  (*n* boys = 13; *n* girls =33)  Markers of Autism Risks in Babies-Learning Early Signs (MARBLES)  High-risk ASD population  2007-2014 | **Phthalates** (MEP, MiBP, MiBP, MBP, MHBP, MBzP, MEHP, MEHHP, MEOHP, MECPP, MCPP, MCOP, MCNP) | Maternal urine  2^nd^, 3^rd^ trimester of pregnancy | *Cognitive development*  MSEL  (3 years)  -  *Autistic-like behaviors*  ADOS  (3 years) | Null associations of average concentrations of phthalate biomarkers during mid-to late pregnancy with both ASD and non-typical development.  MEP only associated with an increased risk of non-typical development in the full sample.  *Adjustment confounders*  Child’s birth year, maternal pre-pregnancy body mass index, homeownership as a proxy of socioeconomic status. | No sex-specific effect observed for phthalates prenatal exposure and increased risk of ASD.  Boys: MEP, MBzP, MCPP, MCNP, and DEHP positively associated with non-typical development risk. |
| Sotelo-Orozco et al. (63)  US  Cohort | N = 148 mother-infant dyads  (*n* boys =93; *n* girls=55)  N ASD = 36  Markers of Autism Risks in Babies-Learning Early Signs (MARBLES)  High-risk ASD population  2008-2019 | **Phenols** (BPA, BPS, Triclosan, 2,4-dichlorophenol, 2,5-dichlorophenol, Benzophenone-3)  **Parabens** (MEBP, PRPB)  **Phthalates** (MBP, MBzP, MCNP, MCOP, MCPP, MECPP, MECPTP, MEHHP, MEHHTP, MEOHP, MEP, MHBP, MiBP) | Children urine  (3-6 months) | *Cognitive development*  MSEL  (3 years)  -  *Autistic-like behaviors*  ADOS  (3 years) | No overall association between children EDCs exposure at 3–6 months and ASD or Non-Typical Development risk.  Higher MEP concentrations associated with lower MSEL scores on motor skills and visual perception; higher DEHTP concentrations associated with poorer visual reception and lower composite scores.  Higher concentrations of DEHTP, methyl-paraben, MCNP, and BPA associated with lower ASD risk in single-compound analyses.  *Adjustment confounders*  Child’s birth year, the child’s sex,  maternal age, maternal pre-pregnancy BMI, and parental homeownership | No sex-specific effects discussed |
|  |  |  |  |  |  | (*Continued*) |
|  |  |  |  |  |  |  |
| Uldbjerg et al. (62)  US  Cohort | N = 195 children  (*n* boys =108; *n* girls=87)  Preconception Environmental exposures And Childhood health Effects (PEACE)  General population  2008-ongoing | **Phenols** (BPA)  **Phthalates** (MEP, MBP, MiBP, MBzP, MCPP  MCOP, MCNP, DEHP, MEHP, MEHHP, MEOHP, MECPP) | Maternal/paternal preconception urine  Maternal urine  1^st^, 2^nd^, 3^rd^ trimester of pregnancy | *Autistic-like behaviors*  SRS  (6-15 years) | No maternal or paternal preconception exposure or maternal prenatal exposure to phthalates or BPA associated with children SRS scores.  Inverse, but non-significant associations observed for maternal preconception MiBP and MCOP concentrations and paternal preconception MiBP concentrations, associated with lower SRS T-scores.  During pregnancy, only MBzP showed a positive but non-significant association with SRS scores.  *Adjustment confounders*  Parental age, parental educational status, parental body mass index (BMI) pre-pregnancy. | Inverse, non-significant and no clear sex-specific effects observed.  Boys: higher maternal preconception levels of MBP, MiBP, MBzP, MCPP, and MCOP and paternal preconception levels of MBP, MiBP, and MBzP tended to be associated with lower SRS scores, while MEP and MCPN showed weak positive associations.  Girls: non-significant associations between maternal preconception MBP and MCPP and paternal MiBP levels with higher SRS scores. |
| Yu et al. (59)  CHINA  Cohort | N = 3220  (*n* boys =1645; *n* girls= 1575)  Ma'anshan birth cohort (MABC)  General population  2013-2014 | **Phthalates** (MMP, MEP, MBP, MHWP, MBzP, MEHP, MEOHP, MEHHP) | Maternal urine  1^st^, 2^nd^, 3^rd^ trimester of pregnancy | *Autistic-like behaviors*  CHAT-A  (1.3-5 years)  CABS  (5-6 years)  -  *ADHD* and *emotional and behavioral problems*  C-ASQ; SDQ  (3-6 years) | Higher exposure to MMP, co-exposure to low molecular weight phthalates (MMP, MEP, MBP), and high molecular weight phthalates (MEHP, MEOHP, MEHHP) across pregnancy associated with increased autistic traits.  High MBP, MMP, MEP, MBP, MEHP, MEOHP, MEHHP concentrations associated with increased emotional and behavioral problems.  MEP and MBzP exposure associated with peer relation problems.  *Adjustment confounders*  Maternal age, pre-pregnancy body mass index, education, ethnicity, occupation, annual income, primipara, smoking/drinking during gestation, maternal metabolic risk factors | Associations between MMP, LMWP, DEHP, and total phthalate mixture and autistic traits observed in boys but not in girls, although sex was not a significant effect modifier.  Boys: conduct problems were associated with MBP and total phthalates; hyperactive behaviors associated with MEP.  Girls: emotional problems associated with MMP and MMP, MEP, MBP exposure in girls; hyperactive behaviors associated with MBP. |
| **Abbreviations**: ADI-R: Autism Diagnostic Interview-Revised, ADOS: Autism Diagnostic Observation Schedule, ASQ: Age & Stage Questionnaire, BASC-2: Behavioral Assessment System for Children (2nd Edition), BP1,2,3,8: bisphenol-1, bisphenol-2, bisphenol-3, bisphenol-8, BPA: bisphenol A, BPAF: bisphenol AF, BPB: bisphenol B, BPF: bisphenol F, BPP: 4,4'-(1,4-phenylenediisopropylidene) bisphenol, BPS: bisphenol S, BPZ: bisphenol Z, BUPB: brominated flame retardants, BuPB: butyl paraben, CHAT: Checklist for Autism in Toddlers, CHAT-A: Checklist for Autism in Toddlers (Part A), DDT: dichlorodiphenyltrichloroethane, DEHP: di(2-ethylhexyl) phthalate, ETPB: ethyl paraben, Et-FOSAA: 2-(N-methyl-perfluorooctane sulfonamido) acetate, HCB: hexachlorobenzene, HMW phthalates: high molecular weight phthalates, LMW phthalates: low molecular weight phthalates, MBP: monobutyl phthalate, MBzP: monobenzyl phthalate, M-CHAT: Modified Checklist for Autism in Toddlers, MCHHP: mono(2-ethyl-5-hydroxyhexyl) phthalate, MCHP: mono-cyclohexyl phthalate, MCNP: monocarboxyisononyl phthalate, MCOP: monocarboxyoctyl phthalate, MECPP: mono(2-ethyl-5-carboxypentyl) phthalate, MEHHP: mono(2-ethyl-5-hydroxyhexyl) phthalate, MEHP: mono(2-ethylhexyl) phthalate, MEOHP: mono(2-ethyl-5-oxohexyl) phthalate, MEP: mono-ethyl phthalate, MEPB: methyl paraben, Me-FOSAA: 2-(N-ethyl-perfluorooctane sulfonamido) acetate, MIPP: mono-isopentyl phthalate, MMP: mono-methyl phthalate, MNP: monoisononyl phthalate, MOP: mono-n-octyl phthalate, MCPP: mono(3-carboxypropyl) phthalate, MSEL: Mullen Scales of Early Learning, OC: organochlorine, PFAS: perfluoroalkyl substances, PFDA: perfluorodecanoate, PFDoDA: perfluorododecanoate, PFHxS: perfluorohexane sulfonate, PFNA: perfluorononanoate, PFOA: perfluorooctanoate, PFOS: perfluorooctane sulfonate, PFUA: perfluoroundecanoate, PRPB: propyl paraben, SCQ: Social Communication Questionnaire, SDQ: Strengths and Difficulties Questionnaire, SRS: Social Responsiveness Scale, TCC: triclocarban, TCS: triclosan, WPPSI-IV: Wechsler Preschool and Primary Scale for Intelligence (4th Edition), WISC: Wechsler Intelligence Scale for Children. | | | | | | |
